# Mr. Armstrong, on Purulent Ophthalmia

**Published:** 1809-11

**Authors:** William Armstrong

**Affiliations:** Richmond


### To the Editors of the Medical and Phyfical Journal.

Gentlemen,

THE principles of no art or science demand more deliberate discussion, than that which intimately regards the welfare of our fellow-creature. It is on this account that I have

---

I have witnessed, with great satisfaction, the liberality and judgment with which the controversial department of the Medical and Physical Journal has been conducted. With this impression, I offer a few observations upon a paper published in the Edinburgh Journal, by Mr. W. Simmons, of Manchester. I am induced to make these observations, because the opinion which that gentleman attempts to controvert, is well supported by facts, and points out a preventive for a disease, occasionally most destructive to the organ of vision. I hope also to throw out a few additional hints, illustrating the subject.

Mr. Simmons's paper is entitled, " A Case of purulent Ophthalmia, occurring in an aged person, with remarks on the origin of the disease."

From the symptoms related by the author, it is not difficult to discern, that it was a violent case of that species of purulent ophthalmia, which has been introduced into this country from Egypt, and is now prevalent in Lancashire. I am the more confirmed in this opinion, because, on a late visit to Manchester, I was informed, that a man in the same ward, who attended upon the patient in question, caught the disease from him, and lost an eye.

The observations which the author hath grafted upon this case, and which apply almost exclusively to that species of purulent sore eye, which occurs in new-born infants, demand more attention, on account of their tendency. In these observations, Mr. S. attempts to combat an opinion * which is entertained by Mr. Ware, and many other practitioners of eminence, that the purulent ophthalmia of infants is frequently produced by fluor albus, existing at the time of birth in the mother, and affecting the child's eyes in passing through the vagina. This opinion is founded principally upon the frequent coincidence of these diseases, which are therefore considered by many practitioners, as standing in the relation of cause and effect to each other. In opposition to this opinion, Mr. S. states his ideas in the following words. " My own conviction therefore is, that the ophthalmia occurring in a child, whose parent, at the time of its birth, was affected with leucorrhœa, is merely a *coincidence* of *circumstances in themselves wholly unconnected,* rather than the result of *cause* and *effect;* and consequently, that the *supposed*

* Since writing these remarks, I find, by referring to Mr. Ware's pamphlet, and a paper by Mr. Gibson, of Manchester, published in the Edinburgh Journal, that Mr. Gibson first made this opinion public, although Mr. Simmons gives the credit of it entirely to Mr. Ware.—Quere, Had Mr. Simmons never read Mr. Gibson's paper?

*posed origin* of ophthalmia purulenta is *purely hypothetical.*" After most attentively considering the terms of Mr. Simmons's conviction, and comparing them with the context, I still find great ambiguity in them, and am almost induced to suppose, that an error of the press has crept into this passage of his paper. For who can imagine, in any disease, *a coincidence of circumstances in themselves wholly unconnected?* What author has stated that the purulent ophthalmia (occurring in any way whatever) is *the result of both cause and effect?*—What advocate for the opinion, which Mr. S. combats, has contended, that the *supposed origin* of the ophthalmia purulenta from fluor albus, is otherwise than *purely hypothetical?* In my opinion, it possesses the greatest hypothetical purity, because it is well grounded in fact, and strongly supported by analogy. But, according to Mr. Simmons's mode of reasoning, the opinion is *purely hypothetical* for an opposite reason, viz. because it is not supported by fact. Mr. S. in fact, takes it for granted, that *purely hypothetical* and *ill-founded* are synonymous terms, and therefore considers the hypothesis demolished. By what arguments its destruction is supposed to have been affected, I shall now proceed to examine.

Mr. Simmons's first argument is this: "Within my own observation, the purulent ophthalmia has occurred where the existence of leucorrhœa *was denied.*" Mr. S. will, in candour, admit, that female delicacy might lead to concealment, and that a denial of its presence was not sufficient proof of the non-existence of the disease. But admit, though the disease did not exist at the time, has any author (I would ask) asserted, that leucorrhœa is the only cause of purulent ophthalmia?

Mr. Simmons's second argument is this; that a medical friend, who delivers from 30 *to* 50 *women* annually, had only met with 2 *or* 3 cases of purulent ophthalmia in his *whole* practice; yet *many* of his patients had the fluor albus upon them *preceding delivery.* It would have been more to the point, had Mr. S. been at liberty to state, that his friend's patients had fluor albus *at the time of delivery.* But admitting that this gentleman's patients were affected with fluor albus at the time of delivery, what author has ever imagined, that every child which passes through a vagina affected with fluor albus, will necessary have the purulent ophthalmia induced? Does every inoculation of vaccine or variolous matter produce the cow-pox and small-pox? Is every impure coitus followed by chancre or gonorrhœa?

Mr.

Mr. Simmons's third argument is this; that in Roscoe's case (the case published by Mr. S.), doubtless the disease had a different origin, for it is impossible to suppose, that at such an advanced age, an acute disease could have sprung from, or been at all influenced by such a cause." Doubtless, this aged man could not be born again; and it is even improbable, that the discharge of leucorrhœa had been conveyed by his fingers to his eyes. And it is more than probable, that the disease under which he laboured, was the Egyptian Ophthalmia, and consequently its origin is no longer doubtful.

Mr. Simmons's fourth argument, is an admission for the sake of argument. "But admitting, says he, for the sake of argument, that the discharge of fluor albus had possessed a property adequate to the alleged effect; the increased mucus discharge, preceding parturition, would dilute and carry off any former collection of this noxious fluid; and the vagina greatly shortened, undergo a process similar to ablution, by the dribbling, or sudden flow of the liquor amnii, *after the bursting of the membranes*, so as to render the opinion less and less probable." This explanation of the removal of a noxious discharge is very mechanical, and very unsatisfactory. Every practitioner must be sensible, both how small a quantity of infectious matter is sufficient to produce a fluid of its own nature, and how much that matter may be diluted, and still retain the power of contamination; that the fluid of leucorrhœa is capable of producing a fluid similar in appearance to itself, cannot be doubted. There are few practitioners who have not known instances of married men, who have repeatedly experienced a transient white mucous discharge from the urethra, at the same time their consorts laboured under fluor albus, yet no suspicion could be entertained of their conjugal fidelity. Excoriations of the glans penis, and gonorrhœa of the prepuce, are effects frequently produced by the same cause. Such are the arguments which Mr. Simmons has brought forth.

I shall now say a word or two with respect to the causes, which in Mr. Simmons's opinion, really produce the purulent ophthalmia, viz. *light, irritability, cold,* and *moisture.* These causes are by no means original, and are liable to this most serious objection, that since almost all infants are exposed to these agents, the disease, instead of being the rare occurrence, which Mr. S. and his medical friend have found it in their practice, should be as frequent a disease as any we are acquainted with, judging from from the universality of its causes. But let us, for a moment, attend to Mr. Simmons's convenient mode of reasoning. In the former part of his paper, he says, " But when we have considered the frequency of that female affection (fluor albus), and the comparative rareness of this species of ophthalmia, we shall, *I believe,* be inclined to doubt the validity of the opinion, (viz. that fluor albus ever occasions purulent ophthalmia): in the latter part of his paper, Mr. S. attributes this comparatively rare disease, to causes as common and universal as any he could possibly have selected. The same observation may be made with respect to a peculiar constitution of the atmosphere; an agent so general and widely diffused, cannot be considered with any degree of probability as the cause of the purulent ophthalmia of infants, a disease, as far as my experience or reading extends, in which no remarkable increase or decrease in frequency has at any time been observed. Had it ever occurred in Manchester, as an infantile epidemic, it is somewhat unfortunate that Mr. S's friend, who has been in the habit of delivering from 30 to 50 women annually, should only have met with two or three cases of it.

Upon the whole, it appears to me, that such causes as light, irritability, cold and moisture, as well as occasional injury in the birth, may sometimes induce simple inflammation of the eye; but something more than these is necessary to produce a specific disease.

If to any of my readers I have appeared to be unnecessarily minute in discussing a matter of opinion, respecting the cause of a disease, I beg leave to add, that in attempting to erase any impression which Mr. S's arguments may have made, I have been governed by this conviction, that his remarks may tend to induce some practitioners to neglect those simple means of prevention, which were recommended by Mr. Gibson, in his paper, on a frequent cause of the diseasse in question. The effectual ablution of the child's eyes immediately after birth, &c. in case of concomitant leucorrhœa, is so easy and inoffensive a measure, that I trust Mr. Simmons himself will not fail to recommend it in his practice, although he may still feel convinced that the supposed origin of the ophthalmia purulenta, is purely hypothetical.

I am, &c.

WILLIAM ARMSTRONG.

*Richmond, Oct.* 9, 1809.

